# Extended long-term follow-up of metastatic melanoma patients treated with immunotherapy: late relapses and second primary melanomas

**DOI:** 10.3389/fonc.2023.1241917

**Published:** 2023-12-04

**Authors:** David R. Minor, Kevin B. Kim, R. Krishna M. Karuturi, Mohammed Kashani-Sabet

**Affiliations:** ^1^ California Pacific Medical Center Research Institute and Center for Melanoma Research and Treatment, San Francisco, CA, United States; ^2^ Center for Health Science Research, Sutter Health, Walnut Creek, CA, United States

**Keywords:** follow-up, melanoma, immunotherapy, relapse, survival

## Abstract

**Background:**

Immunotherapy has revolutionized the treatment of patients with advanced melanoma as well as other cancers. Most studies, whether of interleukin-2 or checkpoint inhibitor therapies, have limited follow-up after 5 years, making the incidence of late relapses uncertain. In addition, the incidence of second primary melanomas in patients with stage IV melanoma treated with immunotherapy has rarely been reported.

**Methods:**

We performed a single-institution retrospective study of stage IV melanoma patients treated with interleukin-2 or checkpoint inhibitors over the period from 1992 to 2013. We found 59 patients alive and in remission 5 years after the beginning of immunotherapy and reviewed their subsequent clinical course.

**Results:**

This 59-patient cohort had a median follow-up of 13.1 years, with 36 patients followed up for at least 10 years. Four patients (6.8%) had relapses of their metastatic melanoma at 5, 8, 15, and 17 years after starting the successful immunotherapy. Three of the four are still alive. Only one patient in 690 patient-years of observation had a second primary invasive melanoma.

**Conclusion:**

Although late relapses after immunotherapy for melanoma do occur, we can conclude that the prognosis of stage IV melanoma patients in continuous remission 5 years after starting immunotherapy is excellent, with a progression-free survival of approximately 85% and a melanoma-specific survival of approximately 95% at 20 years in our series. Our incidence of second primary melanomas is lower than usually reported. These results have important implications regarding the follow-up of stage IV melanoma patients successfully treated with immunotherapy.

## Background

Immunotherapy has revolutionized the treatment of patients with advanced melanoma as well as other cancers. It is now common for patients with advanced melanoma to have durable complete remissions lasting many years, and disease progression after 5 years seems uncommon. Most studies, however, have only limited follow-up after 5 years, so the incidence of late relapses is uncertain.

In a study of 270 melanoma patients treated with high-dose bolus interleukin-2 reported by the Cytokine Working Group in 1999 (1), there were only 12 patients who were progression-free 3 to 9 years after beginning therapy, and none of those 12 patients had relapsed during that time period, with a median follow-up of 62 months and a maximum follow-up of 108 months. A recent Danish study (2) demonstrated a 6% 5-year progression-free survival among 464 patients treated with first-line decrescendo continuous-infusion high-dose interleukin-2. They had three relapses in 22 patients with between 5 and 10 years of follow-up but no data on outcomes after 10 years. Richards (3) reported on 84 patients treated with sequential biochemotherapy with a median follow-up of 51 months. He had no relapses in his nine patients in remission at 52 months, but the maximum follow-up reported was only 72 months.

For checkpoint inhibitor therapy, only a few studies have a median follow-up of over 5 years. Schadendorf (4) published survival outcomes on 1,861 patients enrolled in 12 studies of melanoma patients treated with ipilimumab. He found 254 patients to be alive at 3 years with an apparent plateau of the survival curve of 22%; his longest follow-up was 9.9 years. Wolchok (5) reported on the 6.5-year follow-up of a 945-patient three-arm randomized trial comparing ipilimumab and nivolumab as single agents with the combination of both agents. The combination arm had a melanoma-specific survival of 57% at 5 years and 56% at 6.5 years. The study had a minimum follow-up of 77 months and a maximum of 87 months. The 7.5-year follow-up (6) of patients who were progression-free at 3 years in the same trial showed a progression-free survival rate >68%, an overall survival rate >85%, and melanoma-specific survival of 95% at 7.5 years, indicating appreciable relapses between 3 and 7.5 years, without an equal effect on survival. Loo et al. (7) reviewed the experience at Memorial Sloan Kettering Hospital and found 151 patients to be alive 5 years after the initiation of their first immune checkpoint therapy. The median follow-up of their survivors was 93 months, and only 2.6% of their patients experienced progression after 5 years, with no cases of progression in visceral organs. Loo et al. (8) also reviewed eight clinical trials of anti-PD-1 therapy for melanoma, but only one had median a follow-up of over 5 years, and late relapses were not addressed in that review. Robert reported the results of 834 patients treated with pembrolizumab or ipilimumab, but with reported survival of only up to 5 years (9).

Recently, there has been considerable interest in the cost-utility of expensive immunotherapy for patients with melanoma or other cancers. Published studies (10) are currently forced to estimate hazard ratios for progression-free and overall survival for patients after 10 years in the absence of actual published data for that time period. Given that the average melanoma patient might have a life expectancy of over 25 years if cured by therapy, accurate real data on the long-term outcome of patients can be very important for these calculations—for example, the study by Baker (11) used estimated progression-free survival curves showing at least a 30% relapse rate for the combination ipilimumab–nivolumab immunotherapy for the period between 10 and 20 years, whereas in reality the relapse rate may be much lower, increasing the utility of the treatment. The analysis of the cost–benefit of cancer treatments based on immature survival data may create large errors which underestimate the true benefit (12). Better understanding of the incidence of late relapses will also inform decisions regarding appropriate routine imaging studies in patients who have been disease-free for over 5 years.

At our center, we began treating a substantial number of stage IV melanoma patients with interleukin-2-based immunotherapy in 2003, and in 2007 we began participation in clinical trials of checkpoint inhibitor immunotherapy. Here we present a retrospective study of stage IV melanoma patients whom we identified as being in continuous remission for 5 or more years after immunotherapy. Although a comparison of the results between high-dose interleukin-2, biochemotherapy, ipilimumab, and anti-PD-1 therapy might be interesting, the number of patients at our institution in each category is low, many patients received multiple therapies, and much patient data is unavailable due to the use of paper records prior to 2014. Thus, the focus of our study is limited to the 59 patients who survived in complete remission for more than 5 years after starting immunotherapy. We were concerned about both late relapses and the incidence of new primary melanomas in this patient population. Our observation of a few late relapses gives some indication of the frequency of this unfortunate occurrence.

## Methods

After obtaining a waiver of consent from our institutional review board, we conducted a single-institution retrospective analysis of patients treated with immunotherapy from 1992 to 2013. We reviewed the available records of 432 stage IV cutaneous melanoma patients treated with immunotherapy prior to 2014. This included 18 patients treated with high-dose interleukin-2 between 1988 and 2003, 254 patients treated with a combination of interleukin-2 and chemotherapy (biochemotherapy) between 2002 and 2013, and 160 additional patients treated on clinical trials of ipilimumab or nivolumab from 2007 to 2013. Many patients who had initial therapy with biochemotherapy after 2007 received ipilimumab or nivolumab when they progressed or relapsed. One patient received talimogene laherparepvec (TVEC) in addition to ipilimumab. The biochemotherapy consisted of six cycles of inpatient temozolomide, cisplatin, vinblastine, interleukin-2, and alpha-interferon as described by O’Day (13). Responding biochemotherapy patients also received monthly inpatient 42-h infusions of interleukin-2 after biochemotherapy (14). Because we included only patients who started therapy prior to Food and Drug Administration’s approval of anti-PD-1 agents, only 11 patients, all previously treated, received nivolumab or pembrolizumab. In general, patients with age over 65 or with brain metastases were not treated with biochemotherapy. However, those poor-risk patients were treated with checkpoint inhibitors beginning in 2008 when those agents became available on clinical trials. During or following their immunotherapy, four patients received surgery for a persistent disease and five patients received surgery for a recurrent disease in the brain, lung, bowel, adrenal, conjunctiva, or nodes. None of these nine patients relapsed beyond 5 years, and the authors feel that surgery for an oligometastatic disease may be useful in selected patients with incomplete responses to immunotherapy. **Table 1** shows a list of the immunotherapy regimens that produced durable remissions. In total, 21 patients received biochemotherapy only and nine patients received only ipilimumab. The other 29 patients received sequentially more than one immunotherapy or the combination of immunotherapy followed by surgery or radiation for a known or suspected persistent disease. A total of 11 patients received second-line anti-PD-1 therapy. Most patients had follow-up CT scans of the body every 3 months for 5 years after receiving immunotherapy for metastatic melanoma, with the interval decreasing to 6 months in some patients after year 3.

**Table 1:** Number of patients receiving a specific immunotherapy treatment, either alone or followed by surgery or radiation, that produced a remission lasting over 5 years.

Biochemotherapy	21
Ipilimumab	16
Nivolumab	5
Pembrolizumab	4
Interleukin-2	2
Biochemotherapy then surgery	3
Ipilimumab then surgery	3
Ipilimumab then radiation therapy	1
Nivolumab then surgery	1
Pembrolizumab then surgery	1
Ipilimumab with intralesional IL-2	1
Ipilimumab with talimogene laherparepvec	1

Most patients received prior therapies before the abovementioned immunotherapy.

Progression-free survival after immunotherapy was defined as the time after the start of a course of immunotherapy that produced a remission that lasted 5 or more years or the time after the last surgery or radiation for a metastatic disease, whichever was later—for example, if a patient was started on immunotherapy in May 2009 but received surgery or radiation therapy for a persistent or recurrent disease in June 2010 with no change in systemic therapy, progression-free survival and overall survival were measured from June 2010. Data cutoff was March 1, 2023. A binomial test was used to test for the difference in the occurrence of new primary melanomas, with a probability of occurrence of 0.0067 in one patient-year observed. We tested for the probability of a number of new primary melanomas to be 0 or 1 (i.e., *p*-value of 1 occurrence) over 690 patient-years observed. Kaplan–Meier plots were drawn using the R-packages “survival”, “ggsurvfit”, “survminer”, and “ggplot2”.

## Results

Following immunotherapy, 59 patients were progression-free for 5 or more years and are the subjects of this report. They have a median follow-up of 13.1 years, with a minimum of 6 years and a maximum of 27 years. A total of 36 patients have been followed for 10 years or more. The median age at diagnosis of stage IV melanoma was 53 years, with a range from 13 to 76.

**Figure 1:**
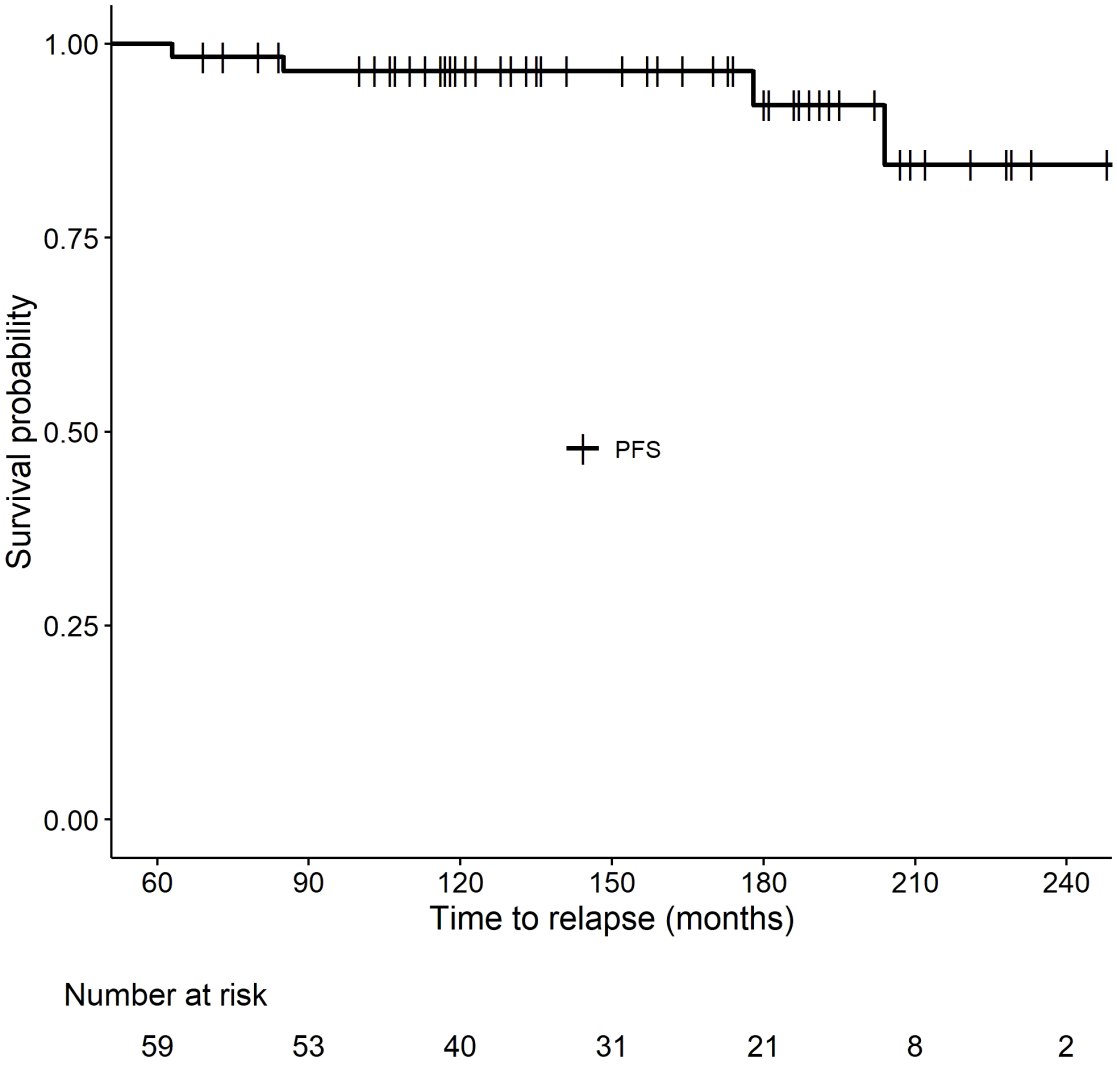
Progression-free survival for 59 patients from 5 to 20 years after immunotherapy.

**Figure 2:**
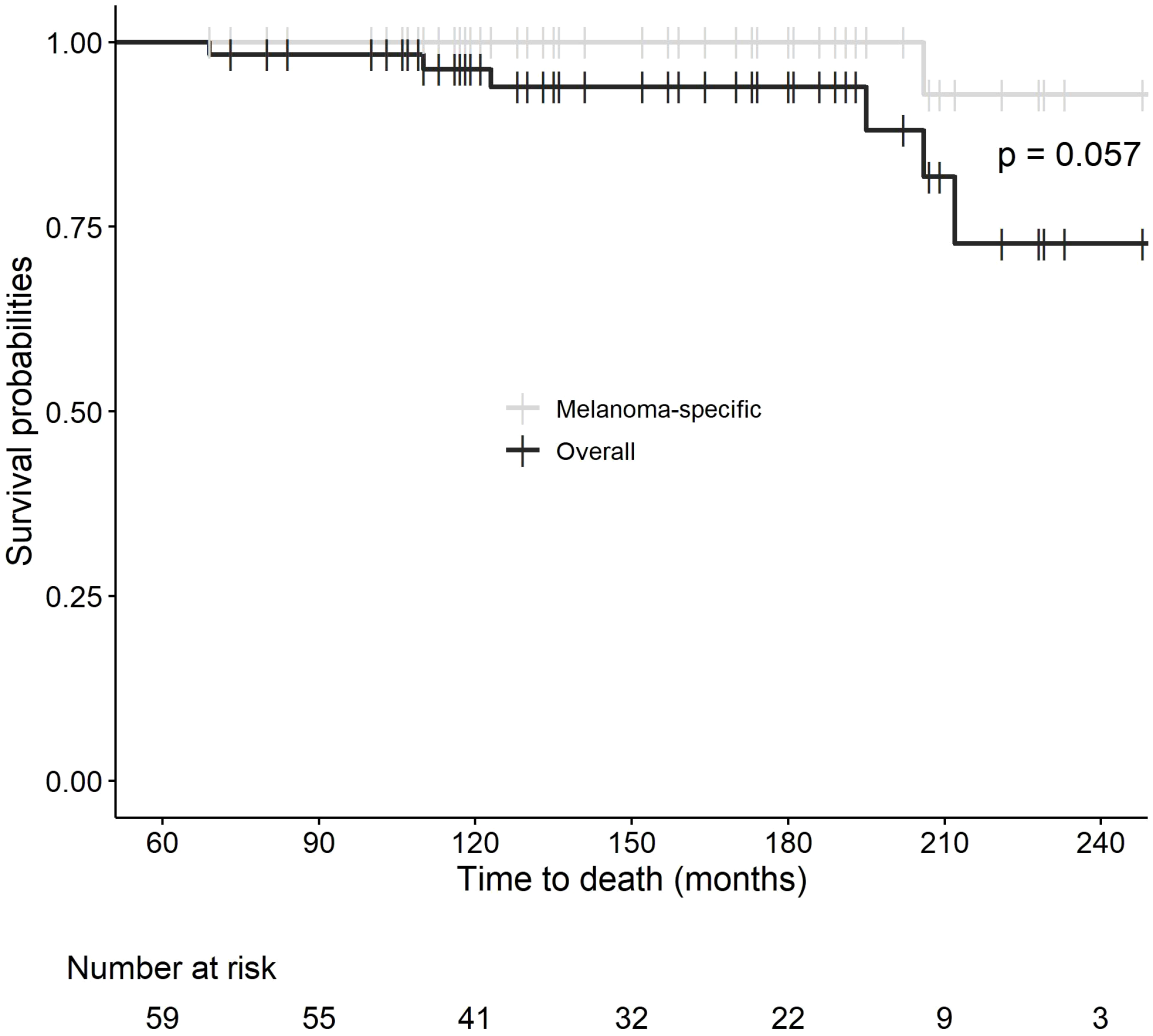
Overall survival and melanoma-specific survival from 5 to 20 years.

In the entire group, only four patients have relapsed (6.8%). One patient treated with biochemotherapy for stage IV melanoma developed multiple brain metastases after 15 years and died 2 years later despite radiation therapy and checkpoint inhibitor therapy. A second patient, also treated with biochemotherapy, developed widespread relapse after 17 years and is alive on therapy with ipilimumab and nivolumab. The third patient, with multiple treatments including biochemotherapy, intralesional interleukin-2, limb perfusion, and ipilimumab had a local relapse after 8.8 years of remission and is alive disease-free 2.5 years later on anti-PD-1 therapy. The fourth patient progressed on biochemotherapy and developed a brain metastasis requiring stereotactic radiation therapy while receiving ipilimumab and then entered complete remission. He had a craniotomy showing radiation necrosis and a microscopic focus of persistent brain metastasis 5.25 years after ipilimumab and stereotactic radiotherapy and is now disease-free 8 years later without further therapy. He is considered to have had a relapse at 5.25 years. His relapse was discovered through a routine MRI brain scan, but in the other three patients the relapses were symptomatic and not found through any routine imaging. Of the 11 patients who received anti-PD-1 therapy with pembrolizumab or nivolumab, none have relapsed so far, although the median follow-up is only 8.7 years in this group. Five patients died from non-melanoma causes, including one patient who died of pneumonia and who had a history of lung irradiation and lung metastases. The other 50 patients are alive and progression-free. Interestingly, in this small series, three of the four patients with late relapses are still alive, with two currently on immunotherapy with anti-PD-1 agents, suggesting that salvage therapy may be successful in this patient population. **Figure 1** shows the progression-free survival of our 59 patients, with approximately 85% progression-free at 20 years. The survival of melanoma patients treated with immunotherapy and disease-free at 5 years is excellent, and their melanoma-specific survival of about 95% is over the next 15 years (**Figure 2**).

Dermatologic histories were available in 54 of our 59 cases. One case of a stage IA second primary melanoma was seen in 690 patient-years of observation in our patients, for a rate of 1.45 cases per 1,000 patient-years of observation. We also observed four new cases of melanoma *in situ*, a diagnosis that is not included in most studies of second primary melanomas but has been reported to occur frequently in the follow-up of initial primary melanomas.

## Discussion

In patients who were disease-free 5 years after starting immunotherapy, we found their long-term survival to be excellent despite occasional relapses. We believe that our median follow-up of 13 years is the longest follow-up of any published study of immunotherapy for stage IV melanoma. Regarding the risk of second primary melanomas, there are variable results in the literature (15). One review (16) gave a cumulative risk of 5.4% at 20 years. The most comprehensive single study was recently published using the Surveillance, Epidemiology, and End Results (SEER) database of 152,811 patients. In that study, Wiener et al. (17) observed 6.7 new primary melanomas per 1,000 patient-years of observation. Our observation of only a single case of a secondary primary melanoma in 690 patient-years of observation indicates that our population had a lower incidence of new primary melanomas, but this finding was of borderline statistical significance (*p* = 0.055, binomial test) when compared with the incidence reported by Wiener. Pennington et al. (18) suggested that checkpoint inhibitor immunotherapy for metastatic melanoma might have a protective effect in regards to secondary primary invasive melanomas, and our data would support that theory. He had an estimated cumulative incidence of second melanomas of 1.3% at 5 years. These findings need further research in a larger group with a careful prospective dermatological follow-up of melanoma patients treated with immunotherapy. We feel that the relatively high rate of secondary *in situ* melanomas may be reflective of the careful follow-up in our patient population.

Viewing the 472 patient-years of follow-up after the 5-year mark, only one case of relapse was found on routine imaging; this was found at 5¼ years in a patient being followed by brain MRI every 6 months after stereotaxic brain radiation. This suggests that routine imaging studies in melanoma patients in remission 5 years after beginning a successful immunotherapy may have limited value.

Our data agree with the study by Loo et al. (8) and show a very low incidence of relapses in melanoma patients treated with immunotherapy and in continuous remission 5 years after beginning immunotherapy as well as excellent overall survival. In contrast to Loo et al., three of our four patients experienced relapses at visceral sites. Our experience also suggests that therapy for these late relapses may be successful. There are several limitations on our study, and we are uncertain as to whether our results can be extrapolated to other melanoma patient populations receiving other immunotherapy regimens. First, the patients who received biochemotherapy received a non-FDA-approved dose schedule of interleukin-2, and their experience may be different from patients treated with high-dose interleukin-2 according to the schedule approved by the Food and Drug Administration in 1998 or other schedules. Second, our study included only a few patients with brain metastases, a population excluded from earlier clinical studies with high-dose interleukin-2 immunotherapy but commonly found in studies of checkpoint inhibitors. Third, our experience with checkpoint inhibitors was largely with single-agent ipilimumab, which, in general, has an inferior short-term outcome (19) compared to anti-PD-1 agents or the combination of ipilimumab and nivolumab. Ipilimumab is now rarely used as first-line single-agent therapy. Fourth, although our series has longer follow-up than other published series, the absolute number of patients is fairly low. Nonetheless, the use of our data on the long-term results with immunotherapy may be more accurate for cost-utility studies than extrapolating survival from short-term trials. Our observation of fewer than expected new primary melanomas in this population needs further study. To our knowledge, the biology underlying late relapses in cancer patients, regardless of therapy, remains poorly understood. Although late relapses after immunotherapy for melanoma do occur, we can conclude that the prognosis of stage IV melanoma patients in continuous remission 5 years after starting immunotherapy is excellent.

## Data availability statement

The raw data supporting the conclusions of this article will be made available by the authors without undue reservation.

## Ethics statement

The studies involving humans were approved by Sutter Health Institutional Review Board (SHIRB@sutterhealth.org). The studies were conducted in accordance with the local legislation and institutional requirements. The ethics committee/institutional review board waived the requirement of written informed consent for participation from the participants or the participants’ legal guardians/next of kin because a retrospective chart review with contact with patients was conducted only to determine survival when the information was not in the chart.

## Author contributions

DM, KK, and MK-S contributed to the conception, design, or planning of the study. DM and RKMK contributed to the analysis of the data. DM, KK, and MK-S contributed to the acquisition of the data. DM, KK, RKMK, and MK-S contributed to the interpretation of the results. DM, KK, and MK-S contributed to the drafting of the manuscript. All authors contributed to the article and approved the submitted version.
